# Reply to the Letter: “Abnormal cerebrospinal fluid composition can accompany CNS involvement in COVID-19”

**DOI:** 10.1055/s-0042-1758379

**Published:** 2022-11-09

**Authors:** Renan Barros Domingues, Fernando Brunale Vilela de Moura Leite, Carlos Senne

**Affiliations:** 1 Senne Liquor Diagnóstico, São Paulo SP, Brazil.

Dear Editor,

We gratefully appreciate the comments by Dr. Finsterer and Dr. Scorza about our paper. 1 However, we believe it is important to point out our disagreements with them.

Dr. Finsterer and Dr. Scorza disagree that the categories stroke, encephalitis, encephalopathy, meningitis, seizures, and headache can be called “syndromes”. A syndrome is a set of medical signs and symptoms that are correlated with each other and often associated with a particular disease or disorder. The word derives from the Greek and means “concurrence”. Only when a syndrome has a definite cause this becomes a disease. 2 Considering that these categories may be linked to different aetiologies and mechanisms, especially in the context of an acute COVID-19, they are true syndromes. Neurologists build their diagnostic reasoning on syndromic, topographic, and etiologic diagnoses. Therefore, the use of the term in our article is not only correct but also makes perfect sense in aiding neurological diagnostic process.

Dr. Finsterer and Dr. Scorza question the non-inclusion of several neurological syndromes in the review. They mention subarachnoid bleeding (SAB), venous sinus thrombosis (VST), cerebral vasculitis, posterior reversible encephalopathy syndrome (PRES), cerebellitis, hypophysitis, opsoclonus myoclonus ataxia syndrome, reversible cerebral vasoconstriction syndrome (RCVS), multiple sclerosis (MS), transverse myelitis, and delirium were not included in the review. First, it is important to note that a review establishes specific search criteria. In our article the criteria were clearly stated. It is possible that other cases with potential cerebrospinal fluid alterations were not included because they did not meet the criteria or because they were outside the search period, which ended in April 2021. Second, Dr. Finsterer and Dr. Scorza certainly know that a diagnosis of multiple sclerosis (MS) could never be made in the context of an acute neurological syndrome associated with COVID-19, since MS is a chronic disease, mostly with a relapsing-remitting course. Our group was the first to present a clinically isolated syndrome (CIS) in a patient with SARS-CoV-2 sequencing in CSF. 3 However, at that time, we were careful in not diagnosing MS, as there would be no way to fill the dissemination in time and space criteria. 4 Therefore, it is not understandable why Dr. Finsterer and Dr. Scorza have included MS in this list. Third, myelitis was included in the review within the context of inflammatory manifestations, such as acute disseminated encephalomyelitis. Our own mentioned article, with the first report mentioned above, was of a myelitis thus reinforcing this idea. 4 It is also unclear why the Dr. Finsterer and Dr. Scorza included delirium in this list. Delirium is a fluctuating, and usually reversible, disturbance of mental function. It is characterized by an inability to pay attention, disorientation, an inability to think clearly, and fluctuations in alertness. Many diseases, medications, and intoxications can cause delirium. The updated nomenclature recommends the term acute encephalopathy to describe a rapidly developing (in less than 4 weeks) pathobiological brain process which is expressed clinically as either subsyndromal delirium, delirium or coma and may have additional features, such as seizures or extrapyramidal signs. 5 Considering the scope of this review of CSF changes associated with acute manifestations of COVID-19, the nomenclature we used seems to be more appropriate than delirium.

Dr. Finsterer and Dr. Scorza mention that it is not comprehensible why the 6 patients with encephalopathy and a polymerase chain reaction (PCR) positive for SARS-CoV-2 were classified as encephalopathy and not as encephalitis. They mention that encephalitis not necessarily goes along with a structural CNS lesion and even the CSF leukocyte count can be normal in these patients. They conclude that it is why the definition of encephalopathy (CNS manifestations of systemic disease in the absence of abnormalities on cerebral imaging) is misleading. These statements indicate that Dr. Finsterer and Dr. Scorza have a rather simplistic notion about encephalitis. First, they cite as a reference a paper on encephalitis with antibodies against the neuronal surface, which essentially affect neuronal function. 6 This has nothing to do with viral encephalitis, in which neuroinvasion and inflammation of the brain parenchyma occur. Second, it is necessary to distinguish the pathological and clinical concepts. From a pathological point of view, true viral encephalitis courses with neuroinvasion, neuroinflammation, and neuronal damage, leading to corresponding neuroimaging and clinical findings. From a clinical point of view the concept is much more difficult. The presence of a positive PCR in CSF can indeed be a defining parameter of a viral encephalitis. This has been demonstrated with HSV virus and herpetic encephalitis and with progressive multifocal leukoencephalopathy due to JC virus. 7 In no other viral encephalitis, despite its clinical utility, the precise sensitivity and specificity of CSF PCR are known. It would be unwise to establish a defining criterion based on a technique whose sensitivity and specificity are not yet known. As we stated in the discussion: “It is not possible to rule out the possibility that at least some of these cases were actually encephalitis rather than encephalopathy cases. The lack of uniformity in case definition between studies may have contributed to such heterogeneity. Distinguishing between COVID-19 encephalitis and COVID-19-associated encephalopathy can be difficult and much other information besides CSF needs to be considered, such as the clinical picture, electrophysiological findings, and the brain magnetic resonance imaging findings. Future studies should establish more homogeneous criteria for distinguishing between these syndromes and for assessing possible differences in neurological prognosis between these two conditions”. Every neurologist who manages these patients will certainly recognize the complexity of these clinical presentations, which are incompatible with conceptual oversimplifications.

Dr. Finsterer and Dr. Scorza declare to be surprised that patients with multisystem inflammatory syndrome in children were excluded from the evaluation and patients with inflammatory syndrome were included and that, in their opinion, is a discrepancy that should be solved. MIS is a rare but serious condition associated with COVID-19, in which different organs are affected, including the heart, lungs, kidneys, brain, skin, eyes, or gastrointestinal organs. There is nothing in common between MIS and the inflammatory syndromes included in the study. The inflammatory syndromes included in the present study present characteristics that overlap with encephalitis and encephalopathies and, therefore, better knowing the CSF findings in these cases brings effective contributions to the clinical practice for those who treat these patients.

Dr. Finsterer and Dr. Scorza state that there is a discrepancy between the total number of patients included ( *n* = 663) and the sum of patients categorised to the seven categories ( *n* = 514). We suggest a careful reading of the following paragraph of Methods, which we believe may contain the answer to Dr. Finsterer and Dr. Scorza: “A total of 663 patients were included in these 75 studies. The clinical diagnoses of CNS syndromes among the patients reported in the studies were the following: hemorrhagic stroke (9 cases; 1.35%), ischemic stroke (16 cases; 2.41%), encephalitis (81 cases; 12.25%), encephalopathy (264 cases; 39.82%), headache (52 cases; 7.84%), other inflammatory syndromes (56 cases; 8.45%), meningitis (4 cases; 0.6%), and seizures (22 cases; 3.32%). The seizure types were described as motor (tonic-clonic) generalized onset seizures (2), focal non motor onset with impaired awareness (2) and unknown (13). The clinical syndrome was not defined by the authors of the studies in 159 cases.” 8 We think it is important to emphasize that this is a review article and that the syndromes were defined by the authors of the original articles and not by us.

Finally, Dr. Finsterer and Dr. Scorza say that it is not comprehensible why Guillain-Barre syndrome (GBS) was excluded. The non-inclusion is perfectly justifiable based on the understanding of the clinical practice of those who assist these patients. SARS-CoV-2 associated with GBS is the same, including its laboratory and therapeutic characteristics, as GBS in general. The CSF parameters of GBS are already well known. The syndromes discussed in this article are very different, they are new situations that were brought into our clinical practice in the context of the COVID-19 pandemic. The clinical approach to such cases has been highly challenging. Among the authors of this article, there are neurologists and clinical pathologists who have been daily dealing with the challenges brought by these cases, identifying, based on this clinical practice, the need for a systematic review about the potential clinical contribution of CSF. Dr. Finsterer and Dr. Scorza also mention a subtype of GBS can go along with brainstem encephalitis (Bickerstaff encephalitis). This is true, and we have some prior experience with this form of encephalitis. However, the article cited by Dr. Finsterer and Dr. Scorza was published in June 2021, 9 therefore not fitting our search criteria.

We must thank again the extensive and detailed comments of Dr. Finsterer and Dr. Scorza. Although they did not bring potential contributions to the improvement of our article, given the conceptual limitations of the comments, they certainly brought a stimulating discussion. The effort to answer each question was rewarded by the opportunity to reinforce the value of our article and by new ideas for possible future studies.
